# Supplementary material for: Generation and Characterization of an Influenza D Reporter Virus
Source: Viruses. 2023 Dec 16;15(12):2444. doi: 10.3390/v15122444 (PMC10747006; doi:10.3390/v15122444)
Supplement: Supplementary file 1 [file viruses-15-02444-s001.zip › SupFigure S1.pdf]

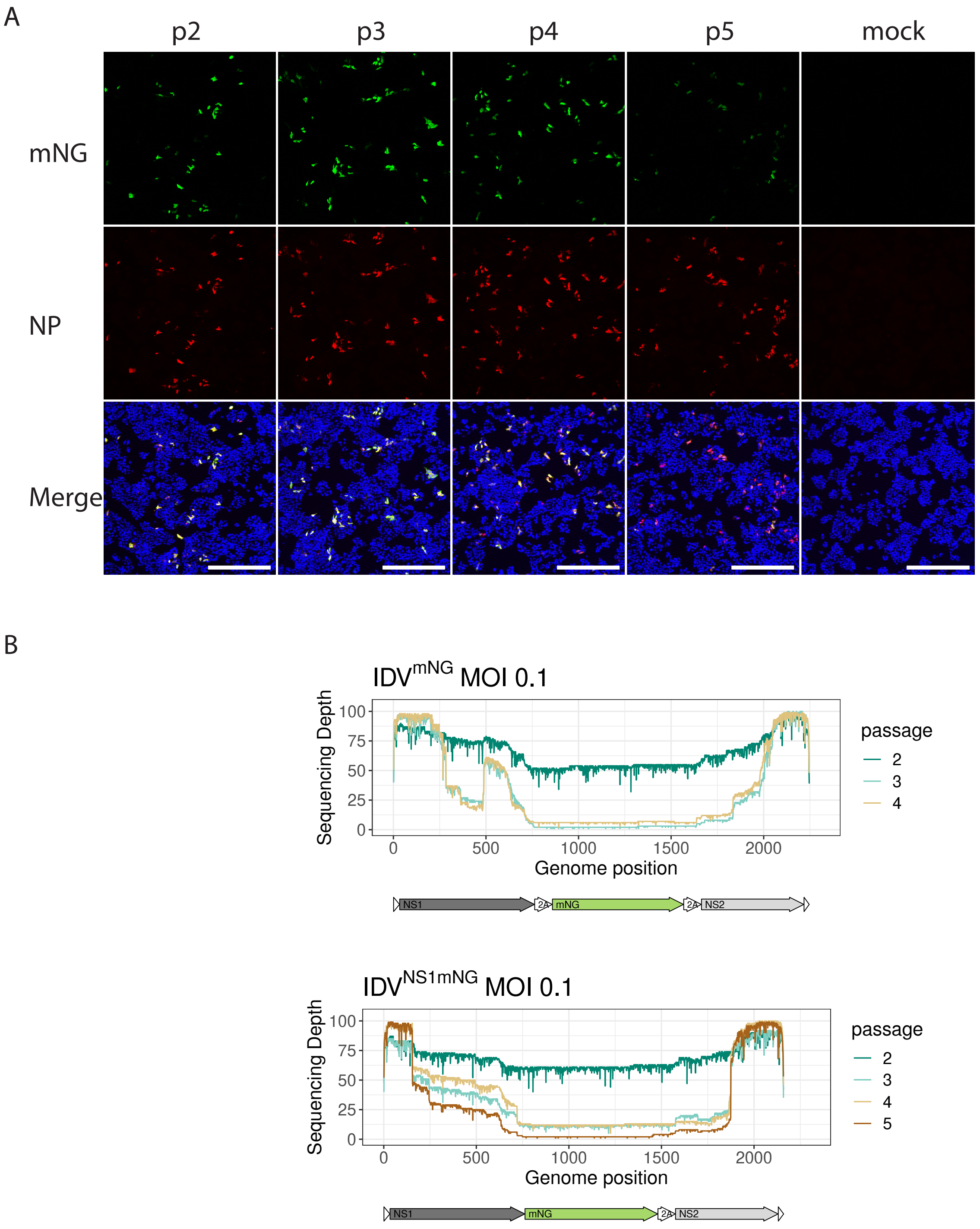

Supplementary Figure S1: mNeonGreen deletions after passaging at MOI 0.1. HRT-18G cells were infected with the passaged IDV^{NS1mNG} and immunostained using an antibody against IDV (anti-NP, red), while mNeonGreen expressing cells are shown in green. Nuclei are stained with DAPI (blue). Scale bar is 500 µm (A). The NS segment of the passaged reporter IDVs was PCR amplified and fully sequenced. The sequencing coverage per nucleotide (y.axis) of the NS segments of IDV (x-axis) after subsequent passages at MOI 0.1 is displayed in colored lines, showing accumulation of deletions at later passages(B).
