# Supplementary material for: Generation and Characterization of an Influenza D Reporter Virus
Source: Viruses. 2023 Dec 16;15(12):2444. doi: 10.3390/v15122444 (PMC10747006; doi:10.3390/v15122444)
Supplement: Supplementary file 1 [file viruses-15-02444-s001.zip › SupFigure S2.pdf]

A

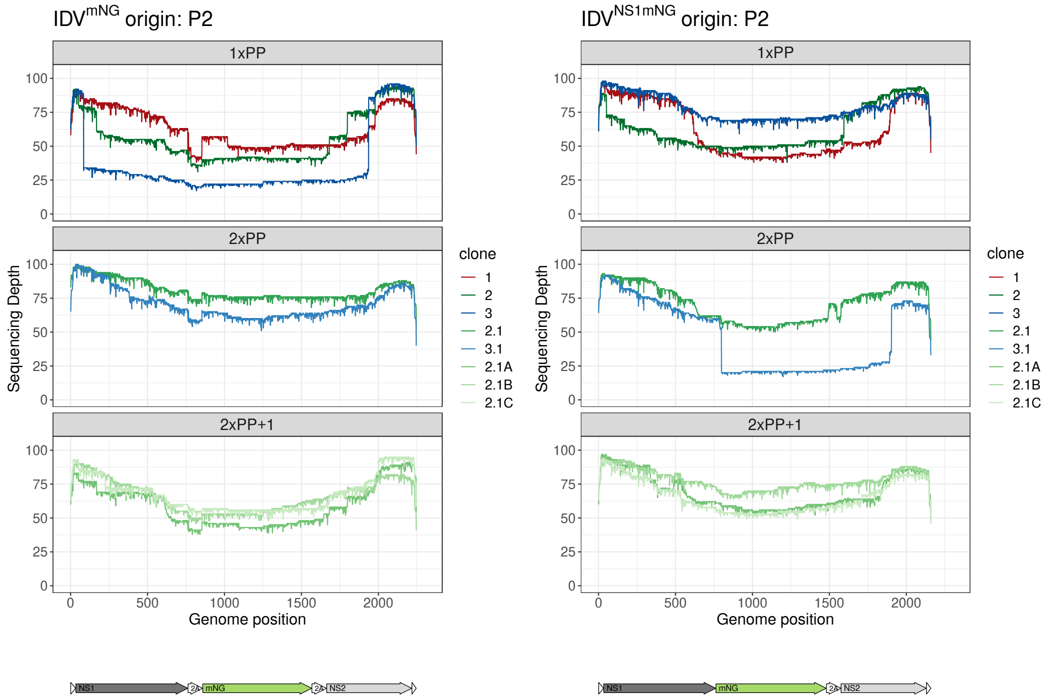

B

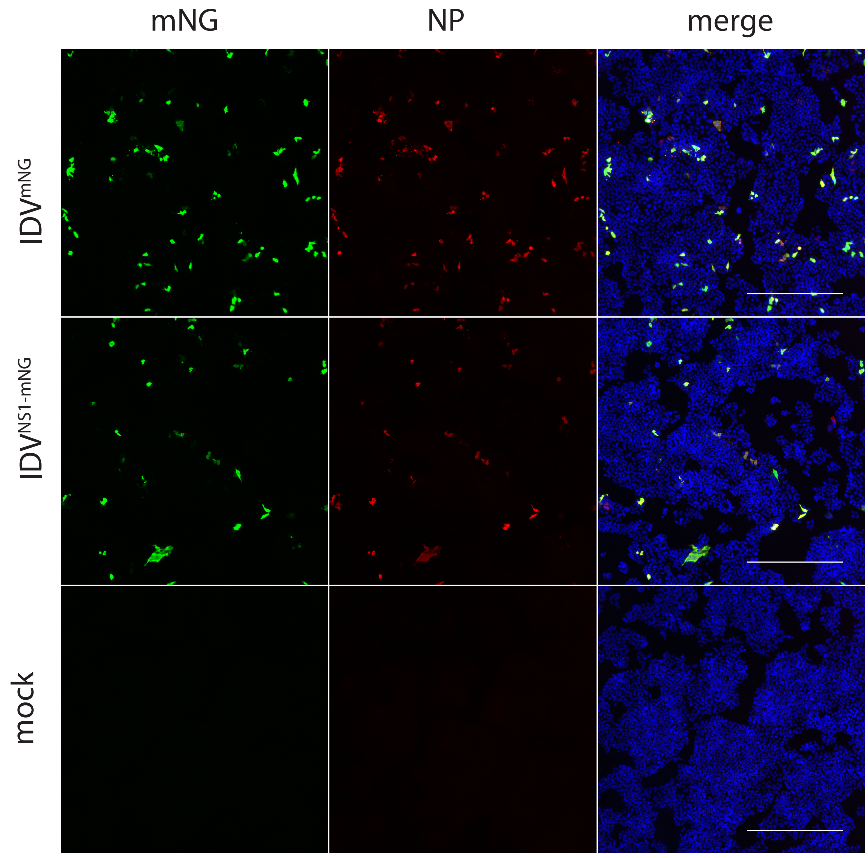

Supplementary Figure S2: Analysis of plaque-purified reporter IDV starting from P2**.** The NS segment of the plaque-purified reporter IDVs was PCR amplified and fully sequenced. The sequencing coverage per nucleotide (y-axis) of the NS segments of IDV (x-axis) after subsequent plaque purification rounds and passages is displayed in colored lines, showing retention of the intact reporter segment over the passages. (1xPP: 1 round of plaque purification and subsequent expansion, 2xPP: 2 rounds of plaque purification and subsequent expansion, 2xPP+1: 2xPP with an additional passage at MOI 0.01). Viral descendants from plaque-purified clone 1 are depicted in shades of red, those descending from clone 2 in shades of green and from clone 3 in shades of blue. (A). HRT-18G cells were infected with the 2x plaque purified and passaged reporter viruses and immunostained using an antibody against IDV (anti-NP, red), while mNeonGreen expressing cells are shown in green. Nuclei are stained with DAPI (blue). Scale bar is 500 µm (B).
